# Supplementary material for: G-actin provides substrate-specificity to eukaryotic initiation factor 2α holophosphatases
Source: eLife. 2015 Mar 16;4:e04871. doi: 10.7554/eLife.04871 (PMC4394352; doi:10.7554/eLife.04871)
Supplement: Supplementary file 1. — List of the plasmids used in this study, their unique lab identifier, lab name, description, PMID of the relevant reference (if available), figure in which they first appear and cognate label in figure legend. DOI: http://dx.doi.org/10.7554/eLife.04871.026 [file elife04871s001.docx]

Table 2: List of the plasmids used in this study, their unique lab identifier, lab name, description, PMID of the relevant reference (if available), figure in which they first appear and cognate label in figure legend

| **ID** | **PLASMID LAB NAME** | ***Description*** | ***Reference*** | ***First appearance*** | ***Label in figure*** |
| --- | --- | --- | --- | --- | --- |
| **75** | pGSTag | Bacterial expression of Glutathione S-transferase with a C-terminally protein kinase A substrate peptide | PMID: 1476738 | 3B | GST |
| **105** | eIF2a-NM_pET30a | Bacterial expression of C-terminally 6XHis tagged human eIF2a (1-185) with A27Q; L46H, V71K solubilizing mutations | PMID:15341733 | 3B | eIF2a |
| **168** | PerkKD-pGEX4T-1 | Bacterial expression of N-terminally GST tagged mouse PERK kinase domain (537–1114) | PMID:9930704 | NA | NA |
| **522** | muPP1G_1-323_pET-30a(+) | Bacterial expression of mouse PP1G (1-323) with no tags | this paper | 5A | PP1 |
| **544** | GST_TEV_muPPP1R15A_539-614 | Bacterial expression of N-terminally GST tagged mouse PPP1R15A (539-614) with TEV cleavage site | this paper | 5A | R15A |
| **571** | GST_TEV_huPPP1R15B_631-701_V1 | Bacterial expression of N-terminally GST tagged human PPP1R15B (631-701) TEV cleavage and an LEHHHHHH C-terminally tag | this paper | 5A | R15B |
| **622** | GST_TEV_muPP1G_1-323 | Bacterial expression of N-terminally GST tagged mouse PP1G (1-323) with TEV cleavage site | this paper | 4E | apo-PP1 |
| **880** | mCherry_N3 | Mammalian expression plasmid encoding mCherry | Clonetech | 1C | mCherry |
| **913** | muPP1G_7-300_pET-30a(+) | Bacterial expression of mouse PP1G (7-300) with no tags | this paper | 2A | PP1 |
| **956** | GST_TEV_muPPP1R15A_539-608 | Bacterial expression of N-terminally GST tagged mouse PPP1R15A (539-608) with TEV cleavage site | this paper | 7E | R15A(539-608) |
| **1044** | GST_TEV_eIF2a-NM_EGFP | Bacterial expression of N-terminally GST tagged and C-terminally EGFP tagged human eIF2a (1-185) with A27Q; L46H, V71K solubilizing mutations | this paper | 5A | eIF2a |
| **1077** | GST_TEV_muPPP1R15A_539-614_W575A | W575A mutation in plasmid 544 | this paper | 6B | W575A |
| **1079** | GST_TEV_muPPP1R15A_539-614_F585A | F585A mutation in plasmid 544 | this paper | 6B | F585A |
| **1080** | GST_TEV_muPPP1R15A_539-614_I589A | I589A mutation in plasmid 544 | this paper | 6B | I589A |
| **1113** | GST_TEV_huPPP1R15B_631-660 | Bacterial expression of N-terminally GST tagged human PPP1R15B (631-660) with TEV cleavage site | this paper | 2B | R15B 631-660 |
| **1162** | GST_TEV_muPPP1R15A_539-614_R571A | R571A mutation in plasmid 544 | this paper | 6B | R571A |
| **1190** | GST_TEV_huPPP1R15B_631-669 | Bacterial expression of N-terminally GST tagged human PPP1R15B (631-669) with TEV cleavage site | this paper | 2B | R15B 631-669 |
| **1191** | GST_TEV_huPPP1R15B_631-684 | Bacterial expression of N-terminally GST tagged human PPP1R15B (631-684) with TEV cleavage site | this paper | 2A | R15B 631-684 |
| **1232** | muPP1G_7-323_pET-30a(+) | Bacterial expression of mouse PP1G (7-323) with no tags | this paper | 3A | PP1 |
| **1250** | FLAG_muR15A_1-653_mCherry | Mammalian expression of N-terminally FLAG and C-terminally mCherry tagged mouse PPP1R15A (1-653) | this paper | 1C | R15A-mCherry |
| **1265** | FLAG_muR15A_1-653_I589A_mCherry | I589A mutation in plasmid 1250 | this paper | 6S1 | R15A(I589A)-mCherry |
| **1298** | FLAG_huR15B_4-713_mCherry | Mammalian expression of N-terminally FLAG and C-terminally mCherry tagged nearly full length human PPP1R15B (4-713) | this paper | 1C | R15B-mCherry |
| **1299** | GST_TEV_huPPP1R15B_631_700_malE | Bacterial expression of N-terminally GST and C-terminally Maltose-Binding Protein tagged human PPP1R15B (631-700) | this paper | 3A | R15B-MBP |
| **1300** | FLAG_muR15A_1-653_W575A_mCherry | W575A mutation in plasmid 1250 | this paper | 6S1 | R15A(W575A)-mCherry |
| **1301** | FLAG_muR15A_1-653_R571A_mCherry | R571A mutation in plasmid 1250 | this paper | 6S1 | R15A(R571A)-mCherry |
| **1302** | FLAG_muR15A_1-653_F585A_mCherry | F585A mutation in plasmid 1250 | this paper | 6S1 | R15A(F585A)-mCherry |
| **1348** | GST_TEV_huPPP1R15B_631_700_R658A_malE | R658A mutation in plasmid 1299 | this paper | 6A | R658A |
| **1349** | GST_TEV_huPPP1R15B_631_700_W662A_malE | W662A mutation in plasmid 1299 | this paper | 6A | W662A |
| **1350** | GST_TEV_huPPP1R15B_631_700_F672A_malE | F672A mutation in plasmid 1299 | this paper | 6A | F672A |
| **1351** | GST_TEV_huPPP1R15B_631_700_I676A_malE | I676A mutation in plasmid 1299 | this paper | 6A | I676A |
| **1368** | eIF2a-NM_R74E_pET30a | R74E mutation in plasmid 105 | this paper | 8B | R74E |
| **1369** | eIF2a-NM_D83K_pET30a | D83K mutation in plasmid 105 (encodes an insoluble protein) | this paper | not shown |  |
| **1370** | eIF2a-NM_E92K_pET30a | E92K mutation in plasmid 105 | this paper | not shown | E92K |
| **1372** | eIF2a-NM_R66E_pET30a | R66E mutation in plasmid 105 | this paper | not shown | R66E |
| **1373** | eIF2a-NM_K86E_pET30a | K86E mutation in plasmid 105 | this paper | not shown | K86E |
| **1374** | muPP1G_7-323_K211E_pET30a | K211E mutation in plasmid 1232 | this paper | not shown | K211E |
| **1375** | muPP1G_7-323_D220K_pET30a | D220K mutation in plasmid 1232 | this paper | 8D | D220K |
| **1384** | GST_TEV_huPPP1R15B_631_695_malE | Bacterial expression of N-terminally GST and C-terminally Maltose-Binding Protein tagged human PPP1R15B (631-695) | this paper | 7D | R15B(631-695) |
| **1388** | muPP1G_7-323_D220K+K211E_pET30a | K211E; D220K mutations in plasmid 1232 | this paper | not shown | K211E/D220K |
| **1389** | eIF2a-NM_R66E+K86E+E92K_pET30a | R66E; K86E; E92K mutations in plasmid 105 | this paper | not shown | R66E/K86E/E92K |
| **1395** | eIF2a-NM_R66E+K86E_pET30a | R66E; K86E mutations in plasmid 105 | this paper | 8C | R66E/K86E |
| **1425** | muR15A_539-614_mCherry | Mammalian expression of C-terminally mCherry tagged core catalytic domain of mouse PPP1R15A (539-614) | this paper | 1C | R15A(539-614)-mCherry |
| **1426** | muR15B_631-700_mCherry | Mammalian expression of C-terminally mCherry tagged core catalytic domain of mouse PPP1R15B (631-700) | this paper | 1C | R15B(631-700)-mCherry |
